# Editorial Comment on Survivorship Postretroperitoneal Lymph Node Dissection for Metastatic Testicular Cancer: A Systematic Review

**DOI:** 10.1111/iju.70141

**Published:** 2025-06-03

**Authors:** Benjamin M. Mac Curtain

**Affiliations:** ^1^ Royal College of Surgeons in Ireland Dublin 2 Ireland

**Keywords:** robotic surgery, rplnd, testicular cancer

1

Calpin et al. authored a systematic review examining retroperitoneal lymph node dissection (RPLND) post chemotherapy, or, as a primary treatment modality in patients with metastatic testicular cancer [1].

This review specifically examined this cohort in relation to sexual, physical, and psychological function post surgery. The systematic review was conducted in a standard fashion; it adhered to PRISMA guidelines and was registered on Prospero, aiding transparency. Eleven studies ranging from the year 2006 to 2022, comprising 1,086 patients, were included, with 80.5% of these patients having Stage 1 disease. There was a large preponderance of nonseminomatous disease (99.2%) within the included data. There were a mixture of open, laparoscopic, and robotic RPLND studies, included.

This review synthesizes evidence that suggests RPLND is, generally, a safe practice, with the side effect profile being mostly non‐severe in the majority of men. However, side effects in general may be more of a worry in this patient cohort due to the younger age of onset of testicular cancer compared to other urological malignancies. Interestingly, in patients who had nerve‐sparing surgery, 88% (unweighted proportion) were found to preserve function to enable antegrade ejaculation.

Overall, long‐term quality of life metrics when compared between RPLND and BEP (bleomycin, etoposide, platinum) were comparable. There was a reported trend that laparoscopic surgery produced more favorable quality‐of‐life metrics postoperatively compared to open surgery.

Possibly, the main takeaway from the above publication is that a shared decision‐making approach must be utilized to inform men undergoing surgery of possible long‐term effects that may decrease their perceived quality of life. It has previously been put forward that high‐volume centers be utilized to try to minimize complications in this cohort [2]. Further studies reporting outcomes of RPLND in seminomatous disease have reported 4 out of 55 patients suffering from long‐term complications [3]. Both of these studies serve as evidence in agreement that, generally, complications are low, but this does not discount the impact it may have on this patient cohort and every effort should be made to minimise the same.

Limitations of the study include the inclusion of retrospective data and the fact that only one of the included studies was a randomized controlled trial. Additionally, outcome reporting heterogeneity between studies limited the author's ability to perform a meta‐analysis. Future prospective studies may be warranted to examine the effect of employing robotic surgery on long‐term patient outcomes and the financial impact on these patients.

Minimizing over treatment is a consideration for all men in this cohort [4]. This review suggests nerve sparing surgery should be attempted where feasible. Care should be taken to carefully explain the risks of surgery and ensure that men are willing to accept the possible decrease in quality of life.

To summarize, this data suggests that, for most men, the procedure is well tolerated in the long term.

## Author Contributions

**Benjamin M. Mac Curtain:** conceptualization, writing – original draft, writing – review and editing, formal analysis, supervision.

## Conflicts of Interest

The author declares no conflicts of interest.
